# Glioma: Application of Whole-Tumor Texture Analysis of Diffusion-Weighted Imaging for the Evaluation of Tumor Heterogeneity

**DOI:** 10.1371/journal.pone.0108335

**Published:** 2014-09-30

**Authors:** Young Jin Ryu, Seung Hong Choi, Sang Joon Park, Tae Jin Yun, Ji-Hoon Kim, Chul-Ho Sohn

**Affiliations:** 1 Department of Radiology, Seoul National University College of Medicine, Seoul, Korea; 2 Center for Nanoparticle Research, Institute for Basic Science, and School of Chemical and Biological Engineering, Seoul National University, Seoul, Korea; 3 Biomedical Research Institute, Seoul National University Hospital, Seoul, Korea; 4 Institute of Radiation Medicine, Seoul National University Medical Research Center, Seoul, Korea; UCSF, United States of America

## Abstract

**Background and Purpose:**

To apply a texture analysis of apparent diffusion coefficient (ADC) maps to evaluate glioma heterogeneity, which was correlated with tumor grade.

**Materials and Methods:**

Forty patients with glioma (WHO grade II (n = 8), grade III (n = 10) and grade IV (n = 22)) underwent diffusion-weighted imaging (DWI), and the corresponding ADC maps were obtained. Regions of interest containing the lesions were drawn on every section of the ADC map containing the tumor, and volume-based data of the entire tumor were constructed. Texture and first order features including entropy, skewness and kurtosis were derived from the ADC map using in-house software. A histogram analysis of the ADC map was also performed. The texture and histogram parameters were compared between low-grade and high-grade gliomas using an unpaired student’s t-test. Additionally, a one-way analysis of variance analysis with a post-hoc test was performed to compare the parameters of each grade.

**Results:**

Entropy was observed to be significantly higher in high-grade gliomas than low-grade tumors (6.861±0.539 vs. 6.261±0.412, *P*  = 0.006). The fifth percentiles of the ADC cumulative histogram also showed a significant difference between high and low grade gliomas (836±235 vs. 1030±185, *P = *0.037). Only entropy proved to be significantly different between grades III and IV (6.295±0.4963 vs. 7.119±0.3165, *P*<0.001). The diagnostic accuracy of ADC entropy was significantly higher than that of the fifth percentile of the ADC histogram (*P* = 0.0034) in distinguishing high- from low-grade glioma.

**Conclusion:**

A texture analysis of the ADC map based on the entire tumor volume can be useful for evaluating glioma grade, which provides tumor heterogeneity.

## Introduction

Gliomas are the most common primary malignant brain neoplasms, ranging in grade from low to high [1]. The accurate grading of gliomas is critical for planning therapeutic strategies, assessing prognosis, and monitoring response to therapy [2], [3].

Currently, diffusion-weighted imaging (DWI) provides tumor characterization and has been used to differentiate high- from low-grade gliomas. A few recent reports have suggested that high-grade gliomas exhibit lower apparent diffusion coefficients (ADCs) [2], [4]–[7] because of higher tumor cellularity. Recently, Kang et al. revealed that the fifth percentile of the cumulative ADC histogram obtained from DWI was the most promising parameter for differentiating high- from low-grade gliomas [5].

However, the fifth percentile of the cumulative ADC histogram reflects a small portion of the tumor. Instead, texture analysis parameters, such as entropy, show the characteristics of the entire tumor and have the advantage of noninvasively quantifying tumor heterogeneity, something that cannot be achieved reliably via simple visual analysis. It is important to assess tumor heterogeneity because tumors with high intratumoral heterogeneity have been shown to have poorer prognosis, which could be secondary to intrinsic aggressive biology or treatment resistance [8].

Although there is no strict definition of the image texture, it is easily perceived by humans and is believed to be a rich source of visual information [9]. Generally speaking, textures are complex visual patterns composed of entities, or subpatterns, that have characteristic brightness, intensity, size, etc. Thus, texture can be regarded as a similarity grouping in an image [10]. The local subpattern properties derived from a computerized texture analysis give rise to the perception of their attributes of the texture as a whole [11].

Until now, some reports have been published regarding tumor heterogeneity in extracranial tumor using CT and MRI texture analysis. In esophageal cancer, B. Ganeshan et al. [12] found that patients who had heterogeneous tumors with low uniformity and high entropy values assessed via CT texture analysis demonstrated poorer survival. Francesca Ng et al. [13] revealed that CT texture features were associated with the 5-year overall survival rate in patients with primary colorectal cancer. Additionally, textural parameters reflecting tumor heterogeneity were associated with tumor metabolism, stage, and prognosis in lung cancer on non-contrast-enhanced CT [14], [15]. Texture analysis has also been used in breast cancer to improve the distinction between benign and malignant lesions using contrast-enhanced MRI [16].

To date, there have been a few reports on glioma grading using a texture analysis of imaging data. Most of these reports were about the application of texture analysis in the characterization of brain tumors, for example, to discriminate glioblastoma multiforme from malignant glioneuronal tumors, as well as metastasis from gliomas [8], [17]–[19]. There are only a few reports about glioma grading using texture analysis. A study by Karoline et al. [20] demonstrated the potential for CT texture analysis in quantifying tumor heterogeneity in gliomas and showed a correlation between tumor heterogeneity and tumor grade. Additionally, Ananda et al. [21] also revealed that textural features extracted on T2-weighted images were highly discriminant between grade I and grade III gliomas.

To the best of our knowledge, there have been no previous reports examining the ADC textural analysis parameters for glioma grading. We hypothesized that these ADC textural analysis parameters could be helpful for glioma grading. Thus, the purpose of our study was to explore the role of the texture analysis of ADC maps based on the entire tumor volume in determining the grade of gliomas and to identify the textural ADC parameter with the best diagnostic accuracy in glioma grading.

## Materials and Methods

This retrospective study was approved by the institutional review board of the Seoul National University Hospital. The institutional review board waived the need for written informed consent from the participants.

### Patient Selection

Eighty-seven patients with astrocytic tumors who had undergone initial MR imaging at Seoul National University Hospital between October 2007 and January 2013 were selected from the radiology report database. Inclusion criteria were as follows: (a) a histopathologic diagnosis of astrocytic tumors according to the World Health Organization (WHO) criteria without oligodendroglial components, and (b) MR imaging performed with DW at the standard b value prior to surgery or chemoradiotherapy. We excluded 47 patients due to the following reasons: (a) inadequate MR imaging quality due to substantial motion or susceptibility artifacts (n = 8), (b) MR imaging performed at 3 T (n = 38) and (c) small size (maximum diameter ≤1 cm) of the tumor to perform texture analysis (n = 1).

A total of 40 patients were included in the study. Among the 40 enrolled patients, 8, 10 and 21 exhibited WHO grade II astrocytomas, III anaplastic astrocytomas, and IV glioblastomas, respectively. Grade III astrocytomas and grade IV glioblastomas were classified as high-grade gliomas, while grade II astrocytomas were grouped as low-grade gliomas.

### Image Acquisition

All MR images were obtained with a 1.5-T MR imager (Signa HDx or HDxt; GE Medical Systems, Milwaukee, WI) with an eight-channel head coil. The imaging protocol included axial T2-weighted fast spin-echo (repetition time (TR)/echo time (TE), 5,000/131 msec; 25 sections; Flip angle (FA), 90°; section thickness, 5 mm; intersection gap, 1 mm; field of view (FOV), 220×220 mm; matrix, 448×256; one acquired signal; echo train length, 16; voxel resolution, 0.5×0.9×5.0 mm) and axial T1-weighted spin-echo (TR/TE, 466/11 msec; FA, 70°; section thickness, 5 mm; intersection gap, 1 mm; FOV, 220×220 mm; matrix, 320×192; voxel resolution, 0.7×1.1×5 mm) or sagittal T1-weighted 3D inversion recovery fast spoiled gradient echo (TR/TE, 10/4.5 msec; FA, 20°; section thickness, 1 mm; intersection gap, 0 mm; FOV, 220×220 mm; matrix, 240×240; voxel resolution, 0.9×0.9×1 mm) sequences with axial and coronal reconstruction.

Echo-planar DW MR imaging (TR/TE, 10,000/63 msec: b = 0 and 1000 sec/mm^2^; 35 sections; bandwidth, 1953 Hz per voxel; section thickness, 3 mm; intersection gap, 1 mm; FOV, 240×240 or 220×220 mm; matrix, 160×160; two acquired signals; voxel resolution, 1.5×1.5×3.0 mm) was performed in the axial plane prior to the injection of the contrast material.

DW MR images were acquired in three orthogonal directions and combined into a trace image. Using these data, ADC maps were calculated on a voxel-by-voxel basis with the software incorporated into the MR imaging unit.

T1-weighted sequences were repeated after the intravenous administration of a single dose of 0.1 mmol per kilogram of body weight and a rate of 4 mL/sec of gadopentetate dimeglumine (Magnevist; Bayer Schering Pharma, Berlin, Germany) or gadobutrol (Gadovist, Bayer Schering Pharma, Berlin, Germany).

### Volume Acquisition/ADC histograms

The MR data for the ADC map were digitally transferred from the PACS workstation to a personal computer, and 2-dimensional (2-D) regions of interest (ROIs) were drawn manually using ImageJ 1.44 software (available at http://rsb.info.nih.gov/ij/) [22]; the ADC values were summated from the 2-D ROIs. Then, our PC-based in-house software (MISSTA - medical imaging solution for segmentation and texture analysis) was used for the quantification of several features and was implemented with a dedicated C++ language with MFC (Microsoft Foundation Classes, Microsoft, Redmond, Wash). MISSTA calculated texture and first order features automatically with the input ROI information. ROIs that contained the entire tumor were drawn in each section of the ADC maps. Tumor boundaries were defined with reference to the high-signal intensity areas thought to represent tumor tissue on the T2WI by one author (S.H.C., a neuroradiologist with eight years of brain MR imaging experience) via visual inspection [23].

Definite cystic, necrotic, or hemorrhagic areas were excluded. For visual inspection, we used strict criteria of “definite” necrosis as a nonenhancing portion on contrast-enhanced a T1-weighted image and similar signal intensity to CSF on T2-weighted and FLAIR images. We did not define necrosis only as a nonenhancing portion because 14–45% of nonenhancing supratentorial gliomas are malignant, and 25–31% of the GBM showed faint or no detectable enhancement [24] because contrast enhancement on conventional MRI only means the disruption of the blood-brain barrier, not neovascularization [25]. The data acquired from each section were summated to derive voxel-by-voxel ADCs for the entire tumor; this was performed using in-house software.

ADC histograms were plotted with ADC values on the x-axis with a bin size of 1×10^–6^ mm^2^/sec, and the percentage of the total lesion volume was calculated by dividing the frequency in each bin by the total number of voxels analyzed on the y-axis. We also performed a cumulative analysis with the ADC histograms in which the cumulative number of observations in all of the bins up to the specified bin was mapped onto the y-axis and was expressed as a percentage.

For the cumulative ADC histograms, the fifth percentile ADC value, which is the point at which 5% of the voxel values that form the histogram are found to the left in the histogram, were generated [5], [26]. Additionally, 3-D height maps of the ADC signal intensity were generated using in-house software for the representative ADC maps for grades II, III and IV, respectively.

### Texture Analysis

Texture analysis via Gray Level Co-occurrence Matrices (GLCM) is a method for extracting second order statistical texture features in the images. In this study, a texture analysis was performed within ROIs on the area of interest, and we used 3 parameters for the quantitative analysis of the summation of the 2-D ROIs, GLCM entropy as well as the skewness and kurtosis of the image histogram.

For ROIs in the ADC map, entropy was determined as a parameter that indicated both intensity and irregularity.
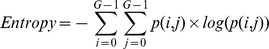



*G* is the number of gray levels used. *P_x_*(*i*) is the *i*th entry in the marginal-probability matrix obtained by summing the rows of *P*(*i, j*). Higher entropy represents increased heterogeneity [27], [28].

### Histopathologic Analysis

The tissue samples were obtained via subtotal/total resection or via image-guided tissue sampling. When performing image-guided stereotaxic biopsy for brain tumors in our hospital, the biopsy-targeted site was determined after preoperative MR imaging; the biopsy was performed at the portion of the tumor that showed the lowest signal on the ADC map and enhancement on the contrast-enhanced T1-weighted image.

Immunohistochemistry was used to measure the Ki-67 labeling index. The routinely used formalin-fixed, paraffin-embedded tissue blocks were sectioned at 4-µm thickness and then used for immunohistochemistry.

The areas with the highest cellularity on inspection were selected, and the Ki-67 labeling index was evaluated using the avidin-biotin complex immunohistochemical technique.

### Statistical Analysis

All statistical analyses were performed with MedCalc software (version 12.6.1.0 for Microsoft Windows 2000/XP/Vista/7; MedCalc Software, Mariakerke, Belgium), SPSS (version 21.0, SPSS Inc., Chicago, Illinois) and GraphPad InStat (version 3.05, 32 bits for Win 95/NT; GraphPad Software, San Diego, CA). Results with P values less than.05 were considered to be significant.

To compare the texture parameters and histogram parameters of high- and low-grade gliomas, the unpaired Student’s t test and receiver operating characteristic (ROC) analysis were applied. Additionally, a one-way analysis of variance with a post-hoc test and ROC analysis were performed to compare the parameters of each grade. The leave-one-out method was used and the McNemar test was performed to compare the accuracies. We used the one-way analysis of variance with a post-hoc test to compare the Ki-67 labeling index of each grade.

After determining the parameter with the highest diagnostic accuracy through the above analyses, we planned to suggest a potential diagnostic algorithm that can differentiate the three different WHO glioma grades. With a Pearson linear regression model, the ADC entropy and the fifth percentile ADC values described above were correlated with the Ki-67 labeling index.

## Results

Table 1 summarizes the ADC texture and histogram parameters of low- and high-grade gliomas. In terms of the comparisons of multiple texture parameters, the entropy and skewness were significantly different between low- and high-grade gliomas. The entropy value was observed to be significantly higher in high-grade gliomas than low-grade tumors (*P = *0.006). Additionally, higher skewness was observed in high-grade gliomas than low-grade tumors (*P = *0.045). No significant difference was found between low- and high-grade gliomas with respect to kurtosis (*P = *0.527). In the cumulative histogram analysis, the fifth percentile of the cumulative histogram showed significant differences between high- and low-grade gliomas (*P = *0.037).

**Table 1:** ADC texture and histogram parameters of low- and high-grade gliomas.

Parameter	Low grade (n = 8)	High grade (n = 32)	*p*-value*
**Texture parameter**			
Entropy	6.261±0.412	6.861±0.539	0.006
Skewness	0.082±0.176	0.582±0.669	0.045
Kurtosis	0.477±1.659	0.945±2.358	0.527
**ADC (x10^−6^mm^2^/sec)**			
Mean	1,327±322	1300±289	0.81
Fifth percentile	1,030±185	836±235	0.037

Note.–Values are the means ± standard deviation.

*Significant difference between two groups (*P<*.05), The difference between two groups was evaluated using the unpaired student’s t-test.

In Table 2, the ADC texture and histogram parameters of the grade II, III and IV gliomas are summarized. Entropy proved to be significantly different between grades II and IV (*P*<0.001) and between grades III and IV (*P*<0.001). Skewness differed significantly between grades II and IV (*P*<0.05) but did not show a significant difference between grades III and IV (*P*>0.05). However, no significant difference was observed between the grade II, III and IV gliomas with respect to kurtosis, mean ADC or fifth percentile ADC value (*P*>0.05).

**Table 2:** ADC texture and histogram parameters of the grade II, III and IV gliomas.

				*p*-value*		
Parameter	Grade II (n = 8)	Grade III (n = 10)	Grade IV (n = 22)	II vs. III	II vs. IV	III vs. IV
**Texture parameter**						
Entropy	6.261±0.4120	6.295±0.4963	7.119±0.3165	>0.05	<0.001	<0.001
Skewness	0.082±0.176	0.265±0.572	0.733±0.671	>0.05	<0.05	>0.05
Kurtosis	0.477±1.659	0.898±2.068	0.967±2.532	0.872**		
**ADC (x10^−6^mm^2^/sec)**						
Mean	1,327±322	1,177±334	1,355±255	0.27**		
Fifth percentile	1,030±185	881±261	815±226	0.088**		

Note.–Values are the means ± standard deviation.

*Significant difference between three groups (*P<*.05); P-values were calculated using a one-way analysis of variance with a post-hoc test.

**P values were calculated using a one-way analysis of variance, and a post-hoc test was not performed.

Table 3 summarizes the results of the ROC analyses of entropy and fifth percentile ADC used to distinguish high- from low-grade glioma. The entropy cutoff value of 6.501 exhibited a sensitivity, specificity and accuracy of 78.1%, 87.5% and 80%, respectively. The fifth percentile ADC cutoff value of 859×10^−6^ mm^2^/sec exhibited a sensitivity, specificity and accuracy of 59.4%, 87.5% and 65%, respectively. There was no significant difference between the diagnostic accuracies of the entropy and fifth percentile ADC (*P = *0.551). However, in the leave-one-out method, entropy showed higher diagnostic accuracy than the fifth percentile of the ADC histogram (72.5% vs. 47.5%, respectively). Additionally, the accuracy of entropy significantly differed from that of the fifth percentile of the ADC histogram (*P = *0.0034).

**Table 3:** ROC results for Entropy and the Fifth percentile of the ADC histogram for glioma grading (low- vs. high-grade).

	Entropy	Fifth percentile ADC
AUC*	0.830 (0.676, 0.930)	0.750 (0.588,0.873)
Sensitivity (%)†	78.1 (25/32)	59.4 (19/32)
Specificity (%)†	87.5 (7/8)	87.5 (7/8)
Accuracy (%)†	80 (32/40)	65 (26/40)
Cutoff value	>6.501	≤859
*P*-value for ROC curve	0.0001	0.0027
*P*-value for the comparison of ROC curves	0.551	

Note.–*The data in parentheses are 95% confidence intervals.

† Sensitivity and specificity for identifying high-grade tumors. The data in parentheses are the numbers used to calculate the percentages.

We performed ROC analyses of entropy for the differentiation between grade III and IV gliomas (Table 4). The entropy cutoff value of 6.792 exhibited a sensitivity, specificity and accuracy of 81.8%, 90% and 84.4%, respectively.

**Table 4:** ROC result for Entropy of ADC for glioma grading (Grade III vs. IV).

	Entropy
AUC*	0.941 (0.861, 1.000)
Sensitivity (%)†	81.8 (18/22)
Specificity (%)†	90 (9/10)
Accuracy (%)†	84.4 (27/32)
Cutoff value	>6.792
*P*-value for ROC curve	<0.0001

Note.–*The data in parentheses are 95% confidence intervals.

† Sensitivity and specificity for identifying grade IV gliomas. The data in parentheses are the numbers used to calculate the percentages.

As entropy displayed the highest diagnostic accuracy for differentiating high- from low-grade gliomas and grade IV from III gliomas, we designed a potential diagnostic algorithm with two cutoff values of entropy. The entropy cutoff value of 6.501 was used to differentiate high- from low-grade gliomas, and the entropy cutoff value of 6.792 was used to identify grade IV gliomas among high-grade gliomas.

**Figure 1:**
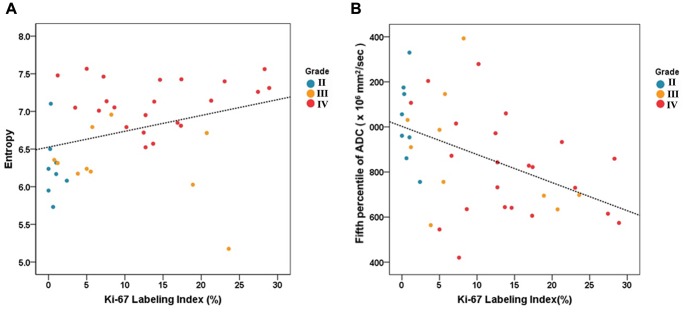
The correlation study of the Ki-67 labeling index (A) with the entropy and (B) with the fifth percentile of ADC using a linear regression model. The relationships were significant. (R2 = 0.1072, *P* = 0.039; R2 = 0.2150, *P* = 0.003, respectively). The tumor grades of the tissue specimens were marked as colored dots (blue = grade II, orange = grade III, red = grade IV).

The relationship between the entropy and Ki-67 labeling index was a significantly positive (R^2^ = 0.1072, *P = *0.039) and the fifth percentile ADC value revealed a significantly negative relationship with The Ki-67 labeling index (R^2^ = 0.2150, *P = *0.003) (Figure 1). The Ki-67 labeling index differed significantly between grade II and grade III (0.689±0.798 vs. 9.349±8.447, *P = *0.030) and between grade II and grade IV (0.689±0.798 vs. 14.117±7.928, *P = *0.0002). However, no significant difference was observed between the grade III and IV gliomas (*P = *0.387).

**Figure 2:**
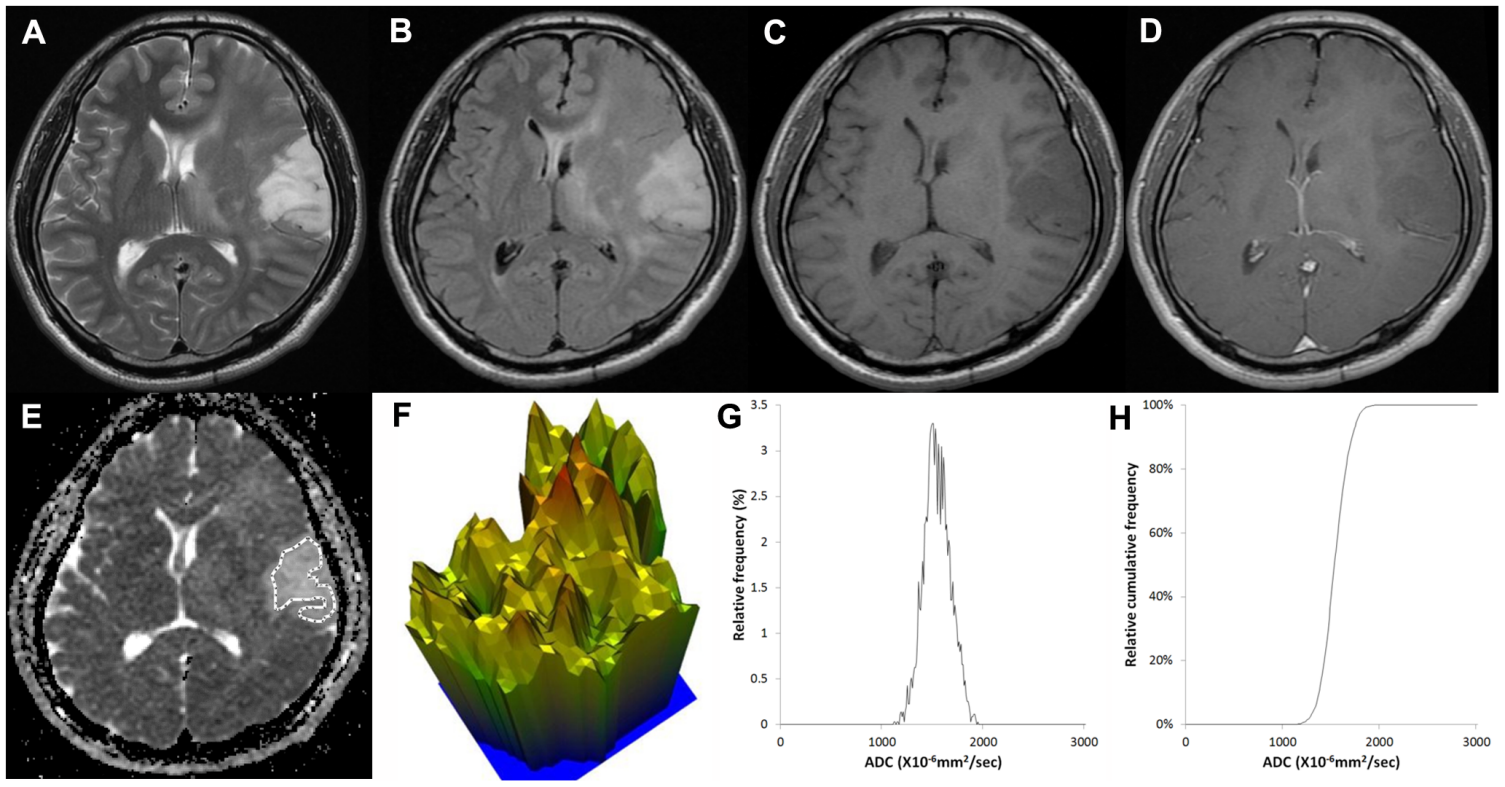
Images of a 43-year-old male with a grade II astrocytoma. (A) T2-weighted image, (B) T2 FLAIR image, (C) T1-weighted image, (D) contrast-enhanced T1-weighted image, (E) ADC map with ROI placement, with the corresponding (E) 3-D height map of the ADC signal intensity, (G) histogram of ADC and (F) cumulative ADC histogram. The entropy value of ADC was 6.168.

**Figure 3:**
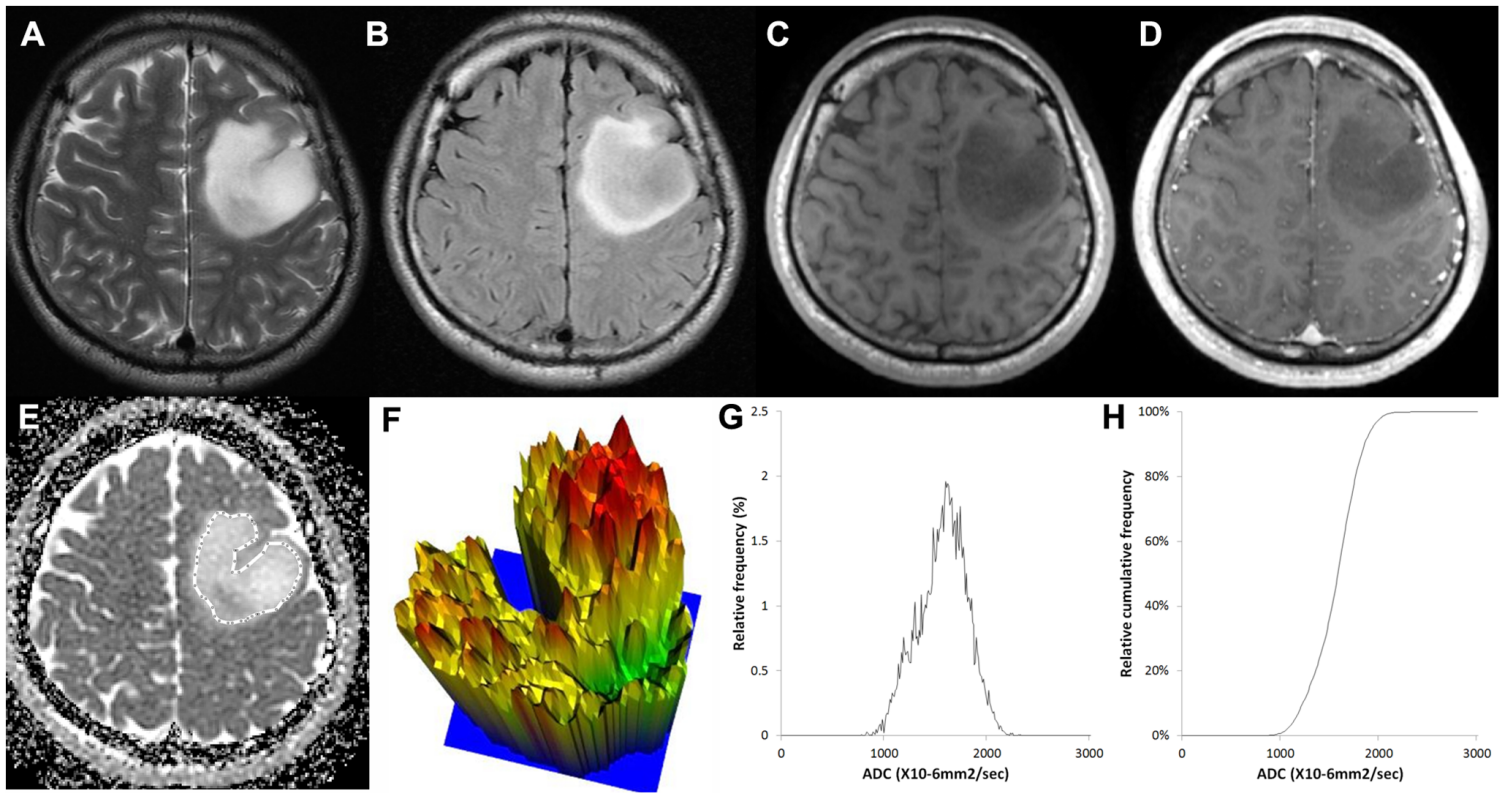
Images of a 30-year-old male with a grade III anaplastic astrocytoma. (A) T2-weighted image, (B) T2 FLAIR image, (C) T1-weighted image, (D) contrast-enhanced T1-weighted image, (E) ADC map with ROI placement, with the corresponding (E) 3-D height map of the ADC signal intensity, (G) histogram of ADC and (F) cumulative ADC histogram. The entropy value of ADC was 6.792.

**Figure 4:**
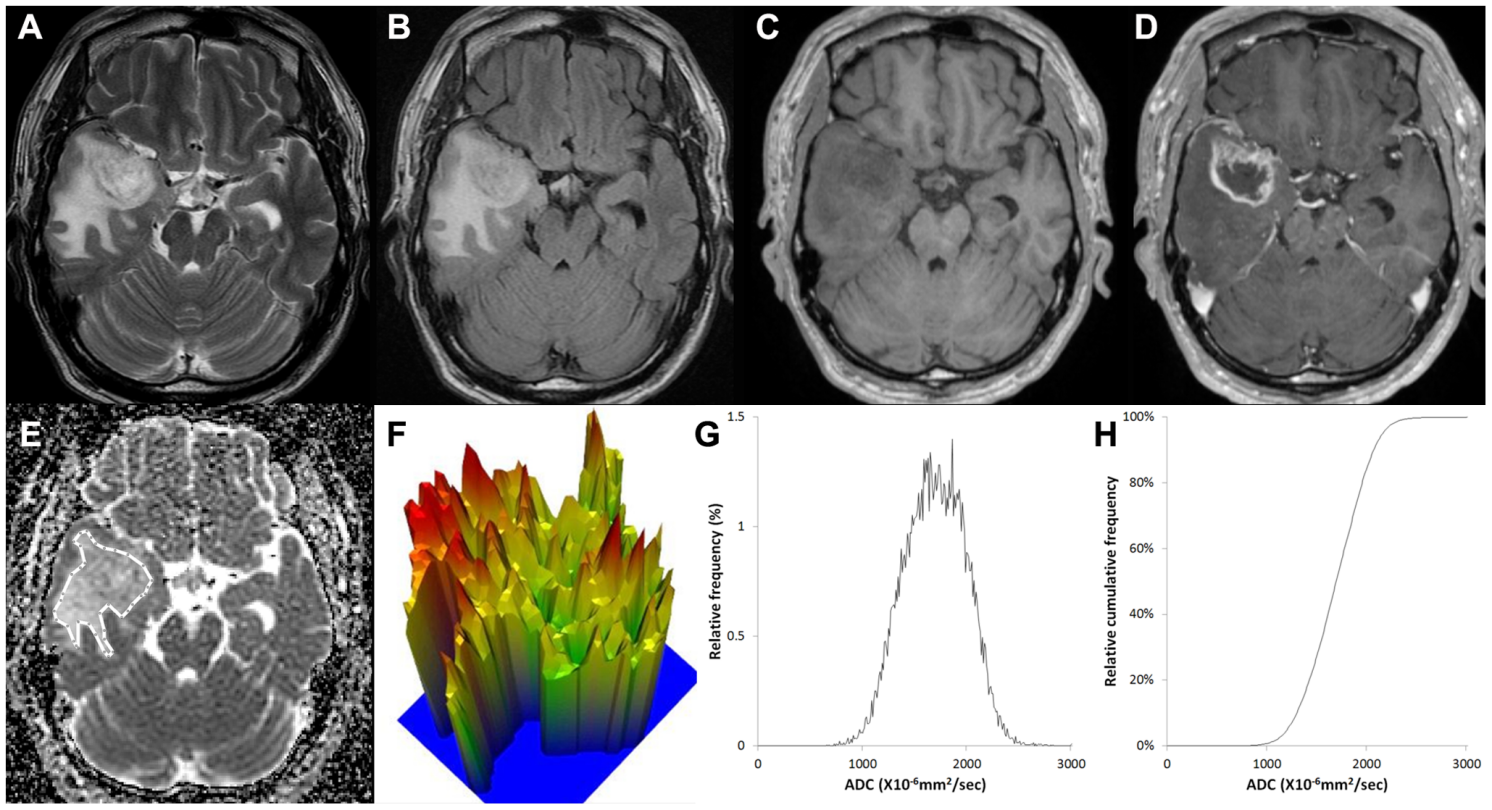
Images of a 62-year-old male with a grade IV glioblastoma. (A) T2-weighted image, (B) T2 FLAIR image, (C) T1-weighted image, (D) contrast-enhanced T1-weighted image, (E) ADC map with ROI placement, with the corresponding (E) 3-D height map of the ADC signal intensity, (G) histogram of ADC and (F) cumulative ADC histogram. The entropy value of ADC was 7.05.

Figures 2, 3 and 4 show representative ADC maps and histograms of grade II, III and IV, respectively. In Figure 2, grade II glioma showed the narrowest spectrum of the ADC signal intensity, exhibited a relatively flat appearance of the 3-D height map of the ADC signal intensity and had a low ADC entropy value (6.168). In contrast, in Figure 4, grade IV glioma showed the widest spectrum of the ADC signal intensity, displayed a rugged appearance in the 3-D height map for ADC signal intensity and had a high ADC entropy value (7.05).

## Discussion

The results of our study suggest that the high entropy, high skewness and low fifth percentile values of the ADC histograms based on the entire tumor volumes could be used to differentiate between high- and low-grade gliomas; the high entropy may also be useful for discriminating grade IV from III gliomas. The diagnostic accuracy of the ADC entropy was significantly higher than that of the fifth percentile of the ADC histogram in distinguishing high- from low-grade gliomas. Thus, we believe that the entropy value from the ADC maps can be used to differentiate between high- and low-grade gliomas, as well as between grade III and grade IV gliomas, which reflects the heterogeneity of tumors.

Tumors are heterogeneous on both the genetic and histopathological levels, with intratumoral spatial variation in the cellularity, angiogenesis, extravascular extracellular matrix, and areas of necrosis^8^; in the present study, these were evaluated using an ADC histogram and texture analysis based on the whole tumors. The utility of DWI and ADC mapping in the pre-operative diagnosis of brain tumors has been examined by several groups with respect to assessing cellularity or grade or predicting tumor response to treatment. DWI and ADC have also been used in distinguishing between enhancing and non-enhancing areas, between tumor and perifocal vasogenic edema, or between viable tumor and necrosis; an inverse relationship between cellularity and diffusivity has been observed in various tumors, including lymphoma, high-grade glioma, meningioma, and medulloblastoma [2], [29]–[34]. However, it is well known that the mean ADC value has limitations in the evaluation of glioma grading and response to treatment due to the heterogeneity of gliomas. Spatial and temporal heterogeneity in the ADC signal is based on the destruction of normal anatomy by tumors, vasogenic edema, tumor cellularity, degenerative changes (hemorrhage, cystic or mucinous degeneration), or the compression of normal structures. Signal changes may be additive or cancel each other out [35]. Additionally, Kang et al. [5] also found that grade IV gliomas showed a higher mean ADC than either grade II or grade III gliomas due to the inclusion of microscopic areas of necrosis and the partial volume-averaging effect of adjacent areas of necrosis. Thus, we postulated that the heterogeneity of ADC values within gliomas can be useful for their grading and found that the difference in entropy between grade III and IV was bigger than that for grade II and III, which indicates that the increased ADC entropy in high-grade glioma can be explained by high heterogeneity.

Additionally, entropy may be representative of entire tumors. In previous studies, the specific value, such as the minimum or fifth percentile of the cumulative ADC histogram, was used to distinguish between high- and low-grade gliomas. However, specific feature values reflect only a small portion of the tumor. Instead, texture analysis parameters, such as entropy, show the characteristics of the entire tumor and have the advantage of noninvasively quantifying tumor heterogeneity. A limited number of texture parameters were described because we eliminated the parameters that were statistically insignificant. We extracted multiple texture features, including entropy, kurtosis, skewness, homogeneity, GLCM moments, GLCM Inverse Difference Moment, GLCM contrast, and variance; we also extracted multiple shape descriptors, including volume, effective diameter, surface area, sphericity, discrete compactness and roundness. Then, after statistical analysis, the parameters that were statistically insignificant were excluded. We selected entropy from among multiple texture analysis parameters because entropy was the most commonly used texture analysis parameter in the previous studies using CT texture analysis and because entropy reflects the heterogeneity the tumor [12]–[15], [27].

In our study, the entropy cutoff value of 6.501 exhibited a sensitivity, specificity and accuracy of 78.1%, 87.5% and 80%, respectively, for distinguishing between high- and low-grade gliomas. The entropy cutoff value of 6.792 exhibited a sensitivity, specificity and accuracy of 81.8%, 90% and 84.4%, respectively, for the differentiation between grade III and IV gliomas; these appear to be comparable with previous studies. Zacharaki et al. revealed that the accuracy, sensitivity, and specificity of the binary Support Vector Machine (SVM) classification were 88%, 85%, and 96%, respectively, for discriminating between high- and low-grade glioma, 55.6%, 90.9% and 75.0%, respectively, for discriminating between grade II and III gliomas and 100%, 90.9% and 96.4%, respectively, for discriminating between grade IV and grade II gliomas. This study required the co-registration of all sequences (T1-weighted image, contrast-enhanced T1-weighted image, T2-weighted image, T2 FLAIR image, relative cerebral blood volume map) and computer-assisted multi-step classification [18]. Additionally, based on T2-weighted imaging, the study of Ananda et. al indicated that contrast, intensity and entropy, kurtosis, and spectral energy showed differences between grade I and grade III; sensitivity, specificity and accuracy were not calculated [21]. We believe that a textural analysis using multimodalities will be more helpful for the diagnosis and grading of gliomas, and future studies are warranted.

Ki-67, a nuclear antigen specific for proliferating cells [36], is used for the evaluation of tumor proliferation, and the positive relationships of Ki-67 with higher cell density and tumor grade have been well-known for the astrocytic gliomas [37]. Some studies showed that the minimum or fifth percentile values of the ADC histogram correlated well with the Ki-67 labeling index, but the relationship between the mean ADC and the Ki-67 labeling index was insignificant in high-grade gliomas [38], [39]. Our results demonstrated that the fifth percentile values of the ADC histogram had a negative correlation with the Ki-67 labeling index in gliomas, including low-grade gliomas, and that entropy had a positive relationship with the Ki-67 labeling index. The elevated Ki-67 labeling index correlates with tumor aggressiveness, and entropy reflects spatial irregularity; these could be said to explain the positive relationship between entropy and the Ki-67 labeling index.

It is well known that oligodendrogliomas have different imaging findings from astrocsytomas. Saito et al. have demonstrated that the mean cerebral blood volumes measured via dynamic susceptibility contrast perfusion imaging of the oligodendroglial tumors were significantly higher than those of the astrocytic tumors, irrespective of tumor grade [40]. Additionally, Cha et al. postulated that the reason for elevated cerebral blood volume in oligodendroglioma compared with other gliomas could be that most oligodendroglial tumors are located in cortical areas and have a direct involvement with gray matter. Because the normal cortical gray matter contains a greater number of blood vessels compared with that of white matter, tumors involving the gray matter may exhibit higher vascular density [41]. Additionally, gliomas with oligodendroglial components are reported to have better clinical outcomes compared with pure astrocytic tumors [42], [43]. Thus, we believe that the gliomas with oligodendroglial components should be dealt as other glioma entities, and future study is warranted for this issue.

In terms of T1 shortening within the glioma, which can affect ADC values, the major factors include hemorrhage and calcification. We excluded gliomas with oligodendroglial components, so hemorrhage could affect the ADC values (a decrease in ADC). We believe that hemorrhage is also one of imaging findings for high-grade glioma (especially for glioblastoma) and makes tumor imaging heterogeneous; thus, we assert that hemorrhage is also helpful for glioma grading.

Apart from the intrinsic limits of any retrospective study, our study has several limitations. First, relatively few patients were included in this study given the enrollment period. We excluded patients who performed MR imaging at 3T or using outside hospital MR scanners to minimize the variation of image quality. The difference in signal intensities on diffusion-weighted images obtained at different magnetic fields is regarded as insignificant because molecular movement is independent from the magnetic field [44]. Thus, a texture analysis of the ADC map on 3T MRI may be similar to this study on 1.5T MRI. Additionally, MR images obtained on 3T enable the increase of the contrast-to-noise ratio between normal brain parenchyma and the infiltrative tumor, which could make it easy to determine tumor boundaries on ADC map. We believe that future study at 3T is warranted for this issue. Only a small number of low-grade gliomas (n = 8) was included; however, it is well known that low-grade gliomas account for 10–15% of all adult primary intracranial tumors [45], which is very similar to our study setting. A further prospective study that includes a larger population is warranted to strengthen the statistical power. Second, the tumor boundary was defined with reference to a high signal intensity on T2WIs, and tumor infiltration as well as peri-tumoral edema was included in the ROIs. However, the differentiation between these two components ‘is impossible in the imaging studies. Third, despite the exclusion of any visible foci of suspected artifacts on the ADC map from the ROI measurements [46], the possibility of including extreme ADCs resulting from DW MR imaging and ADC map misregistration artifacts remains. The texture analysis parameters obtained from the ADC map can be presumed to be less affected by these artifacts and, therefore, appear to be more reliable histogram parameters than the specific percentile of the cumulative ADC histogram (such as the minimum or fifth percentile). Fourth, in this study, the whole tumor was used for texture analysis, and the study did not use the individual phenotypes of enhancement, necrosis and edema/invasion. The differentiations between edema and tumor infiltration and between the nonenhancing solid portion of the tumor and microcystic necrosis are impossible via visual inspection because microcystic necrosis and nonenhancing solid portions showed intermediate signal intensity on T2WI and hypointensity on contrast-enhanced T1WI [24]. However, we believe that the inclusion of microcystic necrosis can represent true microenvironment of the tumor. Fifth, we used the manual segmentation method to define tumor boundaries, which can be labor-intensive and limited by inter- and intra-observer reproducibility.

In conclusion, this study reveals that the entropy of the ADC histogram could be used for distinguishing between high- and low-grade gliomas, as well as between grade IV and III gliomas with diagnostic accuracies of 80% and 84.4%, respectively. We suggest a potential glioma grading schema using two ADC entropy cutoff values for separating the three different WHO glioma grades.

## References

[1] Schwartzbaum JA, Fisher JL, Aldape KD, Wrensch M (2006) Epidemiology and molecular pathology of glioma. Nat Clin Pract Neurol 2: 494–503; quiz 491 p following 516. 10.1038/ncpneuro0289 16932614

[2] Sugahara T, Korogi Y, Kochi M, Ikushima I, Shigematu Y, et al (1999) Usefulness of diffusion-weighted MRI with echo-planar technique in the evaluation of cellularity in gliomas. J Magn Reson Imaging 9: 53–60. 10030650 10.1002/(sici)1522-2586(199901)9:1<53::aid-jmri7>3.0.co;2-2

[3] Daumas-Duport C, Scheithauer B, O’Fallon J, Kelly P (1988) Grading of astrocytomas. A simple and reproducible method. Cancer 62: 2152–2165. 3179928 10.1002/1097-0142(19881115)62:10<2152::aid-cncr2820621015>3.0.co;2-t

[4] Hilario A, Ramos A, Perez-Nunez A, Salvador E, Millan JM, et al (2012) The added value of apparent diffusion coefficient to cerebral blood volume in the preoperative grading of diffuse gliomas. AJNR Am J Neuroradiol 33: 701–707. 22207304 10.3174/ajnr.A2846 PMC8050428

[5] Kang Y, Choi SH, Kim YJ, Kim KG, Sohn CH, et al (2011) Gliomas: Histogram Analysis of Apparent Diffusion Coefficient Maps with Standard- or High-b-Value Diffusion-weighted MR Imaging-Correlation with Tumor Grade. Radiology 261: 882–890. 21969667 10.1148/radiol.11110686

[6] Lee EJ, Lee SK, Agid R, Bae JM, Keller A, et al (2008) Preoperative grading of presumptive low-grade astrocytomas on MR imaging: diagnostic value of minimum apparent diffusion coefficient. AJNR Am J Neuroradiol 29: 1872–1877. 18719036 10.3174/ajnr.A1254 PMC8118927

[7] Murakami R, Hirai T, Kitajima M, Fukuoka H, Toya R, et al (2008) Magnetic resonance imaging of pilocytic astrocytomas: usefulness of the minimum apparent diffusion coefficient (ADC) value for differentiation from high-grade gliomas. Acta Radiol 49: 462–467. 18415792 10.1080/02841850801918555

[8] Davnall F, Yip CS, Ljungqvist G, Selmi M, Ng F, et al (2012) Assessment of tumor heterogeneity: an emerging imaging tool for clinical practice? Insights Imaging 3: 573–589. 23093486 10.1007/s13244-012-0196-6 PMC3505569

[9] Materka A, Strzelecki M (1998) Texture analysis methods–a review. Technical university of lodz, institute of electronics, COST B11 report, Brussels: 9–11.

[10] Rosenfeld A, Kak AC (2014) Digital picture processing: Elsevier.

[11] Levine MD, Levine MD (1985) Vision in man and machine: McGraw-Hill New York.

[12] Ganeshan B, Skogen K, Pressney I, Coutroubis D, Miles K (2012) Tumour heterogeneity in oesophageal cancer assessed by CT texture analysis: preliminary evidence of an association with tumour metabolism, stage, and survival. Clin Radiol 67: 157–164. 21943720 10.1016/j.crad.2011.08.012

[13] Ng F, Ganeshan B, Kozarski R, Miles KA, Goh V (2013) Assessment of primary colorectal cancer heterogeneity by using whole-tumor texture analysis: contrast-enhanced CT texture as a biomarker of 5-year survival. Radiology 266: 177–184. 23151829 10.1148/radiol.12120254

[14] Ganeshan B, Abaleke S, Young RC, Chatwin CR, Miles KA (2010) Texture analysis of non-small cell lung cancer on unenhanced computed tomography: initial evidence for a relationship with tumour glucose metabolism and stage. Cancer Imaging 10: 137–143. 20605762 10.1102/1470-7330.2010.0021 PMC2904029

[15] Win T, Miles KA, Janes SM, Ganeshan B, Shastry M, et al (2013) Tumor Heterogeneity and Permeability as Measured on the CT Component of PET/CT Predict Survival in Patients with Non-Small Cell Lung Cancer. Clinical Cancer Research 19: 3591–3599. 23659970 10.1158/1078-0432.CCR-12-1307

[16] Chen W, Giger ML, Li H, Bick U, Newstead GM (2007) Volumetric texture analysis of breast lesions on contrast-enhanced magnetic resonance images. Magn Reson Med 58: 562–571. 17763361 10.1002/mrm.21347

[17] Eliat PA, Olivie D, Saikali S, Carsin B, Saint-Jalmes H, et al (2012) Can dynamic contrast-enhanced magnetic resonance imaging combined with texture analysis differentiate malignant glioneuronal tumors from other glioblastoma? Neurol Res Int 2012: 195176. 22203901 10.1155/2012/195176 PMC3238409

[18] Zacharaki EI, Wang SM, Chawla S, Yoo DS, Wolf R, et al (2009) Classification of Brain Tumor Type and Grade Using MRI Texture and Shape in a Machine Learning Scheme. Magnetic Resonance in Medicine 62: 1609–1618. 19859947 10.1002/mrm.22147 PMC2863141

[19] Schad LR, Bluml S, Zuna I (1993) MR tissue characterization of intracranial tumors by means of texture analysis. Magn Reson Imaging 11: 889–896. 8371644 10.1016/0730-725x(93)90206-s

[20] Skogen K, Ganeshan B, Good C, Critchley G, Miles K (2013) Measurements of heterogeneity in gliomas on computed tomography relationship to tumour grade. J Neurooncol 111: 213–219. 23224678 10.1007/s11060-012-1010-5

[21] S AR, Thomas T (2010) Texture Description of low grade and high grade Glioma using Statistical features in Brain MRIs. Int J of Recent Trends in Engineering and Technology 4: 27–33.

[22] Abràmoff MD, Magalhães PJ, Ram SJ (2004) Image processing with ImageJ. Biophotonics international 11: 36–42.

[23] Emblem KE, Nedregaard B, Nome T, Due-Tonnessen P, Hald JK, et al (2008) Glioma grading by using histogram analysis of blood volume heterogeneity from MR-derived cerebral blood volume maps. Radiology 247: 808–817. 18487536 10.1148/radiol.2473070571

[24] Fan GG, Deng QL, Wu ZH, Guo QY (2006) Usefulness of diffusion/perfusion-weighted MRI in patients with non-enhancing supratentorial brain gliomas: a valuable tool to predict tumour grading? Br J Radiol 79: 652–658. 16641420 10.1259/bjr/25349497

[25] Maia AC Jr, Malheiros SM, da Rocha AJ, da Silva CJ, Gabbai AA, et al (2005) MR cerebral blood volume maps correlated with vascular endothelial growth factor expression and tumor grade in nonenhancing gliomas. AJNR Am J Neuroradiol 26: 777–783. 15814920 PMC7977110

[26] Tozer DJ, Jager HR, Danchaivijitr N, Benton CE, Tofts PS, et al (2007) Apparent diffusion coefficient histograms may predict low-grade glioma subtype. Nmr in Biomedicine 20: 49–57. 16986106 10.1002/nbm.1091

[27] Ganeshan B, Miles KA, Young RC, Chatwin CR (2007) Hepatic entropy and uniformity: additional parameters that can potentially increase the effectiveness of contrast enhancement during abdominal CT. Clin Radiol 62: 761–768. 17604764 10.1016/j.crad.2007.03.004

[28] Haralick RM, Shanmuga K, Dinstein I (1973) Textural Features for Image Classification. Ieee Transactions on Systems Man and Cybernetics Smc3: 610–621.

[29] Gupta RK, Cloughesy TF, Sinha U, Garakian J, Lazareff J, et al (2000) Relationships between choline magnetic resonance spectroscopy, apparent diffusion coefficient and quantitative histopathology in human glioma. Journal of neuro-oncology 50: 215–226. 11263501 10.1023/a:1006431120031

[30] Guo AC, Cummings TJ, Dash RC, Provenzale JM (2002) Lymphomas and High-Grade Astrocytomas: Comparison of Water Diffusibility and Histologic Characteristics1. Radiology 224: 177–183. 12091680 10.1148/radiol.2241010637

[31] Kono K, Inoue Y, Nakayama K, Shakudo M, Morino M, et al (2001) The role of diffusion-weighted imaging in patients with brain tumors. American Journal of Neuroradiology 22: 1081–1088. 11415902 PMC7974804

[32] Wilke M, Eidenschink A, Müller-Weihrich S, Auer D (2001) MR diffusion imaging and 1H spectroscopy in a child with medulloblastoma. Acta Radiologica 42: 39–42. 11167330

[33] Pauleit D, Langen KJ, Floeth F, Hautzel H, Riemenschneider MJ, et al (2004) Can the apparent diffusion coefficient be used as a noninvasive parameter to distinguish tumor tissue from peritumoral tissue in cerebral gliomas? Journal of Magnetic Resonance Imaging 20: 758–764. 15503327 10.1002/jmri.20177

[34] Castillo M, Smith JK, Kwock L, Wilber K (2001) Apparent diffusion coefficients in the evaluation of high-grade cerebral gliomas. American journal of neuroradiology 22: 60–64. 11158889 PMC7975568

[35] Baehring JM, Bi WL, Bannykh S, Piepmeier JM, Fulbright RK (2007) Diffusion MRI in the early diagnosis of malignant glioma. Journal of neuro-oncology 82: 221–225. 17029014 10.1007/s11060-006-9273-3

[36] Scholzen T, Gerdes J (2000) The Ki-67 protein: from the known and the unknown. J Cell Physiol 182: 311–322. 10653597 10.1002/(SICI)1097-4652(200003)182:3<311::AID-JCP1>3.0.CO;2-9

[37] Kiss R, Dewitte O, Decaestecker C, Camby I, Gordower L, et al (1997) The combined determination of proliferative activity and cell density in the prognosis of adult patients with supratentorial high-grade astrocytic tumors. Am J Clin Pathol 107: 321–331. 9052383 10.1093/ajcp/107.3.321

[38] Higano S, Yun X, Kumabe T, Watanabe M, Mugikura S, et al (2006) Malignant astrocytic tumors: clinical importance of apparent diffusion coefficient in prediction of grade and prognosis. Radiology 241: 839–846. 17032910 10.1148/radiol.2413051276

[39] Sunwoo L, Choi SH, Park CK, Kim JW, Yi KS, et al (2013) Correlation of apparent diffusion coefficient values measured by diffusion MRI and MGMT promoter methylation semiquantitatively analyzed with MS-MLPA in patients with glioblastoma multiforme. J Magn Reson Imaging 37: 351–358. 23023975 10.1002/jmri.23838

[40] Saito T, Yamasaki F, Kajiwara Y, Abe N, Akiyama Y, et al (2012) Role of perfusion-weighted imaging at 3T in the histopathological differentiation between astrocytic and oligodendroglial tumors. European journal of radiology 81: 1863–1869. 21543173 10.1016/j.ejrad.2011.04.009

[41] Cha S, Tihan T, Crawford F, Fischbein NJ, Chang S, et al (2005) Differentiation of low-grade oligodendrogliomas from low-grade astrocytomas by using quantitative blood-volume measurements derived from dynamic susceptibility contrast-enhanced MR imaging. American journal of neuroradiology 26: 266–273. 15709123 PMC7974081

[42] Kanno H, Nishihara H, Narita T, Yamaguchi S, Kobayashi H, et al (2012) Prognostic implication of histological oligodendroglial tumor component: clinicopathological analysis of 111 cases of malignant gliomas. PloS one 7: e41669. 22911839 10.1371/journal.pone.0041669 PMC3404002

[43] van den Bent M, Chinot O-L, Cairncross JG (2003) Recent developments in the molecular characterization and treatment of oligodendroglial tumors. Neuro-oncology 5: 128–138. 12672285 10.1215/S1522-8517-02-00028-5 PMC1920673

[44] Alvarez-Linera J (2008) 3T MRI: advances in brain imaging. European journal of radiology 67: 415–426. 18455895 10.1016/j.ejrad.2008.02.045

[45] Kim H, Choi SH, Kim J-H, Ryoo I, Kim SC, et al (2013) Gliomas: Application of Cumulative Histogram Analysis of Normalized Cerebral Blood Volume on 3 T MRI to Tumor Grading. PloS one 8: e63462. 23704910 10.1371/journal.pone.0063462 PMC3660395

[46] Law M, Young R, Babb J, Pollack E, Johnson G (2007) Histogram analysis versus region of interest analysis of dynamic susceptibility contrast perfusion MR imaging data in the grading of cerebral gliomas. AJNR Am J Neuroradiol 28: 761–766. 17416835 PMC7977348

